# Long-term dietary supplementation with saury oil attenuates metabolic abnormalities in mice fed a high-fat diet: combined beneficial effect of omega-3 fatty acids and long-chain monounsaturated fatty acids

**DOI:** 10.1186/s12944-015-0161-8

**Published:** 2015-12-01

**Authors:** Zhi-Hong Yang, Seika Inoue, Yasuko Taniguchi, Hiroko Miyahara, Yusuke Iwasaki, Jiro Takeo, Hiroshi Sakaue, Yutaka Nakaya

**Affiliations:** Central Research Laboratory, Nippon Suisan Kaisha, 32-3 Nanakuni 1 Chome, Hachioji, Tokyo 192-0991 Japan; Lipoprotein Metabolism Section, Cardio-Pulmonary Branch, National Heart, Lung and Blood Institute, National Institutes of Health, Bethesda, MD 20892 USA; Department of Nutrition and Metabolism, University of Tokushima Graduate School of Health Biosciences, Tokushima, Japan

## Abstract

**Background:**

Pacific saury is a common dietary component in East Asia. Saury oil contains considerable levels of n-3 unsaturated fatty acids (PUFA) and long-chain monounsaturated fatty acids (LCMUFA) with aliphatic tails longer than 18 carbons. In our previous study, consumption of saury oil for 4 to 6 wk improved insulin sensitivity and the plasma lipid profile in mice. However, the long-term effects of saury oil on metabolic syndrome (MetS) risk factors remain to be demonstrated. In the current study, we examined the long-term effects of saury oil on mice fed a high-fat diet, and compared the effect of n-3 PUFA EPA and LCMUFA on MetS risk factor in diet-induced obese mice.

**Methods and Results:**

In Experiment 1, male C57BL/6 J mice were fed either a 32 % lard diet (control) or a diet containing 22 % lard plus 10 % saury oil (saury oil group) for 18 weeks. Although no differences were found in body weight and energy expenditure between the control and saury oil groups, the saury oil diet decreased plasma insulin, non–HDL cholesterol, hepatic steatosis, and adipocyte size, and altered levels of mRNA transcribed from genes involved in insulin signaling and inflammation in adipose tissue. Organ and plasma fatty acid profile analysis revealed that consumption of saury oil increased n-3 PUFA and LCMUFA (especially n-11 LCMUFA) levels in multiple organs, and decreased the fatty acid desaturation index (C16:1/C16:0; C18:1/C18:0) in liver and adipose tissue. In Experiment 2, male C57BL/6 J mice were fed a 32 % lard diet (control), a diet containing 28 % lard plus 4 % EPA (EPA group), or a diet containing 20 % lard plus 12 % LCMUFA concentrate (LCMUFA group) for 8 weeks. EPA or LCMUFA intake increased organ levels of EPA and LCMUFA, respectively. Consumption of EPA reduced plasma lipid levels and hepatic lipid deposition, and decreased the fatty acid desaturation index in liver and adipose tissue. Consumption of LCMUFA decreased plasma non–HDL cholesterol, improved hyperinsulinemia, and decreased the fatty acid desaturation index in adipose tissue. EPA accumulated mainly in liver, and LCMUFA (especially n-11 LCMUFA) accumulated mainly in white adipose tissue, suggesting their possible individual biological effects for improving MetS.

**Conclusion:**

Our results suggest that saury oil-mediated improvement of metabolic syndrome in diet-induced obese mice may possibly be due to a combined effect of n-3 PUFA and LCMUFA.

**Electronic supplementary material:**

The online version of this article (doi:10.1186/s12944-015-0161-8) contains supplementary material, which is available to authorized users.

## Background

Increasing evidence has demonstrated that metabolic syndrome (MetS) is associated with an increased risk of cardiovascular disease, the leading cause of death globally [1]. Diverse factors influence the prevalence of MetS, but the main factors include diet and other lifestyle behaviors [2]. The physiological properties of fatty acids largely depend on the chain length and degree of unsaturation. In general, compared with saturated fatty acid (SFA), polyunsaturated fatty acid (PUFA) and monounsaturated fatty acid (MUFA) improve protection against MetS and cardiovascular disease risk factors [3, 4].

Although fish oil is a common source of long-chain n-3 PUFA including eicosapentaenoic acid (EPA) and docosahexaenoic acid (DHA), some fishes such as saury, pollock, and salmon also have considerable levels of long-chain MUFA (LCMUFA) with aliphatic tails longer than 18 carbons (i.e., C20:1 and C22:1 isomers combined) [5, 6]. We previously reported that ingestion of a concentrate of fish oil–derived LCMUFA improves lipid and glucose homeostasis and adipocyte size in mice [7, 8]. Therefore, examination of the metabolic effects of fish oils rich in both n-3 PUFA and LCMUFA is of great interest. In our previous study, consumption of saury oil for 4 to 6 weeks improved insulin sensitivity and the plasma lipid profile in diet-induced obese mice and type II diabetic mice [9]. However, the long-term effects of saury oil on MetS risk factors remain to be demonstrated. Furthermore, since LCMUFA-rich oil consumption up-regulated mRNA expression of genes related to energy expenditure in diet-induced obese mice [8, 9], estimation of the effect of saury oil on metabolic indices is of great interest.

The aim of this study was to assess the long-term (18 weeks) effect of feeding saury oil to experimentally obese (diet-induced) mice with respect to the plasma parameters for lipid and glucose metabolism, hepatic steatosis, adipocyte size, and resting energy expenditure. Furthermore, to evaluate the independent roles of n-3 PUFA and LCMUFA in saury oil for improving MetS in a separate experiment, the same animal model was used, and mice were fed a high-fat diet supplemented with EPA or LCMUFA ethyl ester for 8 wk. The plasma lipid and glucose homeostasis profiles and the hepatic lipid content were analyzed. Our previous studies have shown that dietary LCMUFA improved MetS risk factors via favourable changes in glucose/lipid metabolism-related gene expression and down-regulation of proinflammatory molecules [7, 8]. In comparison, dietary n-3 PUFA has been shown in various studies to improve MetS through multiple mechanisms, including: reducing hepatic lipid accumulation, decreasing the fraction of atherogenic small, dense LDL, and modulating inflammation and endothelial function [10]. Thus, effects of n-3 PUFA and LCMUFA may be shared and/or complementary. To the best of our knowledge, very few studies have compared the effect of fish oil-derived EPA and LCMUFA on MetS risk factor in MetS animal models. In the current study, we hypothesized that long-term feeding of diet-induced obese mice with saury oil would improve MetS symptoms, and both omega-3 fatty acid and LCMUFA may be major contributing factors with shared and complementary mechanisms involved. Diet-induced obese C57BL/6 mouse was chosen as the experimental model owing to its similarity with common features of human MetS [11].

## Methods

### Materials

Saury oil and fractions of fish oil with given concentrations of ethyl esters of EPA and LCMUFA were produced by Nippon Suisan Kaisha, Ltd. (Tokyo, Japan). Lard was purchased from Romi Smilfood B.V. (Heerenveen, Netherlands). The fatty acid composition of the dietary oils is shown in Table 1.

**Table 1:** Fatty acid composition of the dietary oils incorporated into the diets of the mice

Fatty acids (%)	Lard	Saury oil	EPA	LCMUFA
SFA	33	21	0.0	19.7
14:0	1.5	6.6	0.0	3.5
16:0	25.4	12.5	0.0	12.8
18:0	5.9	1.7	0.0	3.0
20:0	0.2	0.2	0.0	0.4
MUFA	45.8	33.4	0.0	63.9
16:1(n-7)	2.4	5.6	0.0	1.3
18:1(n-9)	43.2	6.3	0.0	4.8
20:1(n-11)	0.0	7.4	0.0	16.8
20:1(n-9)	0.2	2.5	0.0	4.7
22:1(n-11)	0.0	11.0	0.0	34.8
22:1(n-9)	0.0	0.6	0.0	1.5
n-6 PUFA	10.8	2	0.0	0.9
18:2(n-6)	10.8	1.4	0.0	0.8
20:4(n-6)	0.0	0.6	0.0	0.1
n-3 PUFA	1.1	26	96.8	1.1
18:3(n-3)	1.0	1.3	0.0	0.3
20:5(n-3)	0.0	11.1	96.8	0.2
22:5(n-3)	0.1	1.3	0.0	0.2
22:6(n-3)	0.0	12.3	0.0	0.4

### Mice and diets

This study consisted of two experiments. In Experiment 1, twenty male C57BL/6 J mice aged 5 weeks were obtained from Japan SLC, Inc. (Shizuoka, Japan). After acclimatization for a 2-week period, the animals were assigned to one of two dietary groups (*n* = 10 per group) by weight matching. They consumed the experimental diets ad libitum for 18 weeks. Experimental diets were based on a standard purified rodent diet (Research Diets, Inc.) with either lard (control diet; 32 % lard) or saury oil (saury oil diet; 22 % lard and 10 % saury oil) (Table 2). The dose of saury oil in this study was consistent with the dose used in our previous study [9]. At the end of 18 weeks, mice from each group were sacrificed by injecting sodium pentobarbital (300 mg/kg) intraperitoneally without fasting.

**Table 2:** Composition of diets enriched with either lard (control), saury oil, EPA, or LCMUFA fed to mice for 18 or 8 weeks

		Experiment 1	Experiment 2	
Ingredient (g/kg)	Lard	Saury oil	EPA	LCMUFA
Casein	258	258	258	258
L-cysteine	4	4	4	4
Maltodextrin 10	162	162	162	162
Sucrose	89	89	89	89
Cellulose	65	65	65	65
Mineral mixture	13	13	13	13
Vitamin mixture	13	13	13	1.3
Choline bitartrate	3	3	3	3
Soybean oil	32	32	32	32
Experimental oils				
Lard	320	220	280	200
Saury oil	0	100	0	0
EPA	0	0	40	0
LCMUFA	0	0	0	120
Nutritional values				
Energy content, MJ/kg	23	23	23	23
Protein, % of energy	14	14	14	14
Carbohydrate, % of energy	26	26	26	26
Fat, % of energy	60	60	60	60

Experiment 1, mice were fed diets enriched with lard or saury oil for 18 weeks; Experiment 2, mice were fed diets enriched with lard, EPA, or LCMUFA for 8 weeks

In Experiment 2, 5-week-old male C57BL/6 J mice (30 total; Japan SLC, Inc.) were acclimatized for a 2-week period and then assigned to one of three dietary groups (*n* = 10 per group). They consumed the experimental diets ad libitum for 8 weeks. Experimental diets were based on a standard purified rodent diet (Research Diets, Inc.) with either lard (control diet; 32 % lard), EPA concentrate (EPA diet; 28 % lard and 4 % EPA ethyl ester), or LCMUFA concentrate (LCMUFA diet; 20 % lard and 12 % LCMUFA ethyl ester) (Table 2). At the end of 8 weeks, mice from each group were sacrificed by injecting sodium pentobarbital (300 mg/kg) intraperitoneally without fasting.

Body weight and food intake were recorded every week. Retro-orbital bleeds to evaluate plasma lipid profiles were performed under isoflurane anesthesia on mice that had been fasted for 5 h after the acclimation period (0 week) and after 5, 12, and 18 weeks (Experiment 1) and after 4 and 8 weeks (Experiment 2). At the end of the intervention period (18 weeks for Experiment 1 and 8 weeks for Experiment 2), mice were anesthetized with sodium pentobarbital (300 mg/kg) intraperitoneally in the early light phase of the light-dark cycle and blood was taken from the abdominal aorta. Plasma was obtained by centrifugation at 1500 × g for 15 min and stored at −80 °C until further analysis. Mesenteric, epididymal, and subcutaneous white adipose tissue (WAT), liver, duodenum, and muscle were removed, rinsed with PBS, weighed, flash frozen, and stored at −80 °C. The animal protocol was approved by the Animal Care and Use Committee of Tokushima University and was in accordance with guidelines provided by the NIH Guide for the Care and Use of Laboratory Animals.

### Measurement of energy expenditure

In Experiment 1, oxygen consumption and CO_2_ production were measured using the Oxymax system (Columbus Instruments, Columbus, OH, USA) by monitoring individual mice in a metabolic cage with free access to food and drinking water. Oxygen consumption was expressed as mL/kg BW/h, and the respiratory quotient was calculated from the ratio of CO_2_ production/oxygen consumption. Spontaneous locomotor activity was evaluated during a 12-h dark period. Information on motility was measured using the ACTIMO system (Shinfactory, Fukuoka, Japan) by monitoring individual mice with infrared beams.

### Measurement of plasma and organ fatty acid compositions

At the end of each experiment, lipids were extracted from mesenteric, epididymal, and subcutaneous WAT, liver, duodenum, muscle, or plasma followed by methylation, and fatty acid methyl ester (FAME) were quantified with gas chromatography as described [12]. In brief, the total lipids from each organ or plasma were extracted in a 4:1 (v/v) of methanol:hexane solution, followed by methylation with acetyl chloride at 80 °C for 1 hr. Fatty acid (FA) methyl esters were separated using a DB-WAX capillary column (Agilent Technologies) then quantified using gas chromatography (Agilent Technologies). Peaks of FAME were identified by comparison with purified standards Nu-Chek Prep (Elysian, MN, USA) and expressed as the percentage of total fatty acids.

### Plasma biochemical analyses

At a determined time point for each experiment, fasting plasma samples collected in heparinized capillary tubes from the retro-orbital sinus were analyzed for plasma triglyceride (TG), total cholesterol (TC), HDL cholesterol (HDL-C), and glucose concentrations, using a Triglyceride E-Test, a Cholesterol E-Test, an HDL Cholesterol E-Test, and a Glucose CII-Test, respectively (Wako Pure Chemical Industries, Ltd., Osaka, Japan), respectively. Plasma insulin concentrations were determined with an ELISA kit (Morinaga Institute of Biological Science, Inc., Yokohama, Japan).

### Measurement of liver lipid levels

At the end of each experiment, hepatic lipids were extracted from the liver according to Folch et al. [13]. The dried lipid residues were dissolved in 2-propanol containing 10 % (w/w) Triton X-100 for the TG and cholesterol assays. The hepatic TG and TC contents were measured with the Triglyceride E-Test and Cholesterol E-Test, respectively. Furthermore, hepatic lipid deposits in Experiment 1 were visualized by staining with hematoxylin and Oil Red O on 10-μm thick cryosections.

### Determination of adipocyte size

At the end of Experiment 1, distribution of the adipocyte size in Experiment 1 was determined as described [14]. Briefly, adipocytes isolated from epididymal and subcutaneous WAT were fixed with 2 % osmium tetroxide and passed through a 250-μm nylon filter to remove the fibrous tissue. The cells were then washed extensively with isotonic saline. Ten thousand cells were analyzed using the Coulter Multisizer III (Beckman Coulter, High Wycombe, England).

### RNA isolation and real-time PCR

At the end of Experiment 1, total RNA was prepared from epididymal adipose tissue using the QIAzol lysis reagent (Qiagen, Valencia, CA) according to the manufacturer’s instructions, and the RNA concentration was determined by measuring the absorbance at 260 nm. RNA from each sample was assessed for purity by A260/280 ratios, and the integrity of the RNA was verified by agarose gel electrophoresis. Real-time PCR was performed as described [7]. In brief, primers for insulin receptor substrate (*Irs*), insulin receptor (*Insr*), tumor necrosis factor alpha (*Tnf-alpha*), and matrix metallopeptidase 3 (*Mmp3*) are listed in Table 3. Real-time PCR was performed on an 300 Real-Time PCR System (Life Technologies Co., Japan) using SYBR Premix Ex Taq (Takara Bio Inc., Otsu Shiga, Japan), while mouse beta-actin served as the housekeeping gene. The PCR cycling conditions were as follows: 30 s at 95 °C; followed by 40 cycles of 5 s at 95 °C, 34 s at 60 °C; and a final melting curve of 15 s at 95 °C, 1 min at 60 °C, 15 s at 95 °C.

**Table 3:** Oligonucleotide primers used for RNA analysis

Gene	Accession number	Forward primer	Reverse primer
Irs	NM_010570	TCTACACCCGAGACGAACACT	TGGGCCTTTGCCCGATTATG
Insr	NM_010568	ATGGGCTTCGGGAGAGGAT	CTTCGGGTCTGGTCTTGAACA
Tnf-alpha	NM_013693	CAGGCGGTGCCTATGTCTC	CGATCACCCCGAAGTTCAGTAG
Mmp3	NM_010809	ACATGGAGACTTTGTCCCTTTTG	TTGGCTGAGTGGTAGAGTCCC

### Statistical analysis

Data for Experiment 1 were collected at the 0-, 5-, 12-, or 18-week time point, and the Student’s *t*-test was used for statistical analysis of the control versus saury oil treatment group. Data for Experiment 2 were collected at the 4- or 8-week time point, and ANOVA followed by Tukey’s test for multiple comparisons was used to identify significant differences among the control, EPA, and LCMUFA treatment groups. Analyses were performed using Prism software (GraphPad Software, San Diego, CA, USA), and *P* values <0.05 were considered statistically significant. Data are the mean ± SEM.

## Results

### Experiment 1

#### Food intake and body weight

All mice remained healthy throughout the 18-week feeding period. We found no significant differences in food intake (control: 2.6 ± 0.1 g/mouse/day vs. saury oil group: 2.4 ± 0.1 g/mouse/day) or final body weight (control: 43.7 ± 0.6 g vs. saury oil group: 43.4 ± 0.5 g) between the control and saury oil groups (Additional file 1).

#### Metabolic indices

**Fig. 1:**
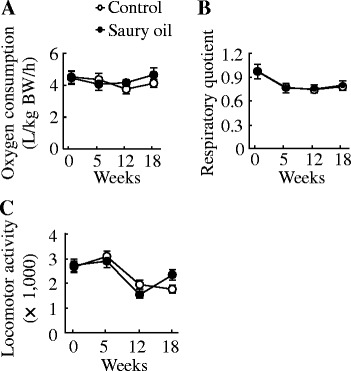
Energy expenditure in diet-induced obese C57BL/6 J mice fed the control or saury oil diet for 18 weeks. Oxygen consumption (**a**), respiratory quotient (**b**) and spontaneous locomotor activity (**c**) at baseline, 5, 12 and 18 weeks. Values are means ± SEM, *n* = 10

Oxygen consumption, respiratory quotient, and spontaneous locomotor activity did not differ between the two diets at the 0-, 5-, 12-, or 18-week time points (Fig. 1). Furthermore, for oxygen consumption, there were no significant differences between each measuring time point in both control and saury oil group. For respiratory quotient, compared with week 0, week 5, it decreased significantly (*P* < 0.001) at week 5, 12, and 18 in either control or saury oil group. For locomotor activity, compared with week 0, it decreased significantly (*P* < 0.001) at week 12 and 18 in either control or saury oil group.

#### Plasma and organ fatty acid compositions

**Fig. 2:**
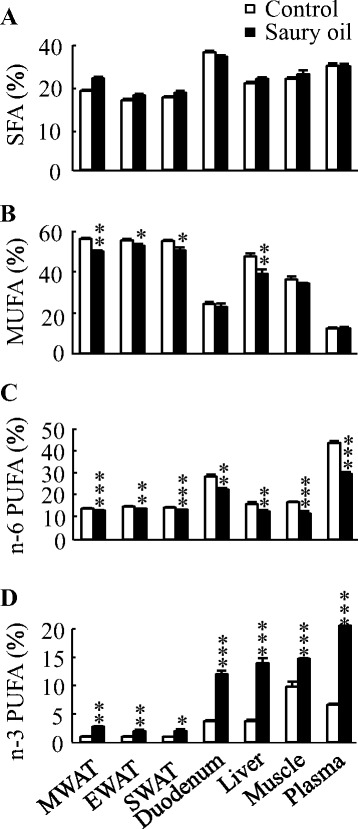
Organ and plasma fatty acid compositions in diet-induced obese C57BL/6 J mice fed the control or saury oil diet for 18 weeks. Percentages of total SFA (**a**), total MUFA (**b**), total n-6 PUFA (**c**), total n-3 PUFA (**d**) in total lipids of WAT, liver, duodenum, muscle, and plasma at the end of 18 weeks. Values are means ± SEM, *n* = 10. *P < 0.05, **P < 0.01, ***P < 0.001 vs. control group. SAF, saturated fatty acids; MUFA, monounsaturated fatty acids; PUFA, polyunsaturated fatty acids; MWAT, mesenteric white adipose tissue; EWAT, epididymal white adipose tissue; SWAT, subcutaneous white adipose tissue

**Fig. 3:**
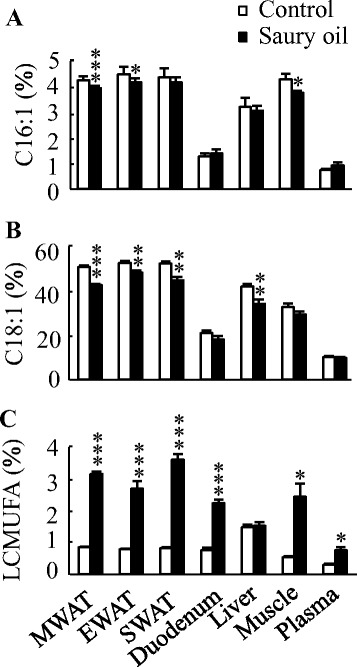
Organ and plasma levels of MUFA in diet-induced obese C57BL/6 J mice fed the control or saury oil diet for 18 weeks. Percentages of palmitoleic acid (**a**), oleic acid (**b**) and LCMUFA (**c**) in total lipids of WAT, liver, duodenum, muscle, and plasma at the end of 18 weeks. Values are means ± SEM, *n* = 10. *P < 0.05, **P < 0.01, ***P < 0.001 vs. control group. MWAT, mesenteric white adipose tissue; EWAT, epididymal white adipose tissue; SWAT, subcutaneous white adipose tissue

**Fig. 4:**
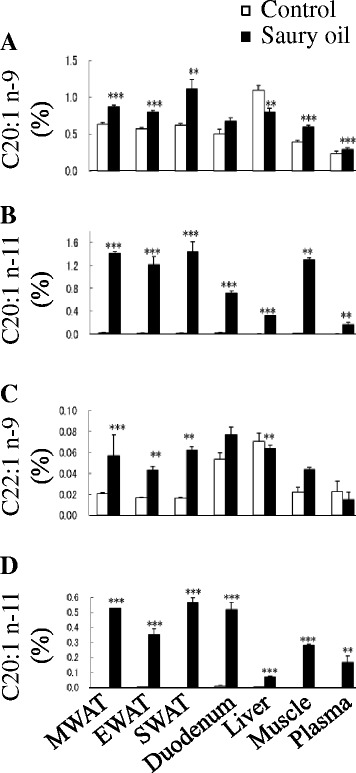
Organ and plasma levels of individual types of LCMUFA in diet-induced obese C57BL/6 J mice fed the control or saury oil diet for 18 weeks. Percentages of C20:1 n-9 (**a**), C20:1 n-11 (**b**), C22:1 n-9 (**c**) and C22:1 n-11 (**d**) in total lipids of WAT, liver, duodenum, muscle, and plasma at the end of 18 weeks. Values are means ± SEM, *n* = 10. **P < 0.01, ***P < 0.001 vs. control group. MWAT, mesenteric white adipose tissue; EWAT, epididymal white adipose tissue; SWAT, subcutaneous white adipose tissue

**Fig. 5:**
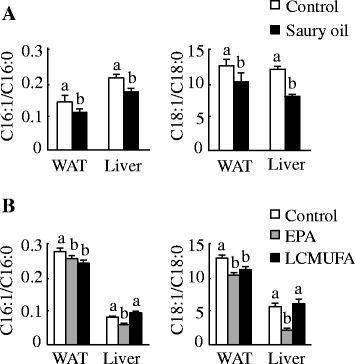
Lipid desaturation index in diet-induced obese C57BL/6 J mice. Ratio of 16:1/16:0 (left panels) and 18:1/18:0 (right panels) in epididymal WAT and liver at the end of 18 weeks in mice fed diets enriched in lard or saury oil (**a**) and at the end of 8 weeks in mice fed diets enriched in lard, EPA, or LCMUFA (**b**). Values are means ± SEM, *n* = 10. Significantly different mean values (*P* < 0.05) are indicated by different lowercase letters. WAT, white adipose tissue

The fatty acid composition in WAT, liver, duodenum, muscle, and plasma reflected the composition of the oils present in the diets (Fig. 2). Although mice fed saury oil had a similar proportion of SFA compared with mice fed the control diet, total n-3 PUFA levels were higher (~0.5- to 3-fold) in each estimated organ/plasma in the saury oil group compared with the control. In contrast, saury oil consumption decreased the percentages of total MUFA (~5–20 %) and n-6 PUFA (~7–32 %) in each estimated organ/plasma. Changes in individual MUFA levels differed according to chain length (Fig. 3). Mice fed the saury oil diet had proportionally more LCMUFAs (~0.1- to 3.6-fold) and less 16:1(n-7) (~7–23 %) and 18:1(n-9) (~7–20 %) than the control diet group in each estimated organ/plasma. For individual types of LCMUFA, as compared with the control group, the changes in n-11 LCMUFA (C20:1 n-11, ~16 to 180-fold; C22:1 n-11, ~6 to 56-fold increase) were larger than than changes in n-9 LCMUFA (C20:1 n-9, ~0.2 to 0.8-fold; C22:1 n-9, ~0.4 to 2.8-fold increase) in each estimated organ/plasma (Fig. 4). Furthermore, our data showed that saury oil consumption suppressed fatty acid desaturation indexes 16:1/16:0 by ~7–15 % (*P* < 0.05) and 18:1/18:0 by ~11–33 % (*P* < 0.05) in WAT and liver (Fig. 5a), along with decreases in 16:1 and 18:1 levels.

#### Plasma glucose and lipid metabolism

**Fig. 6:**
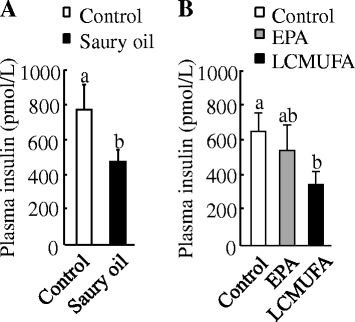
Plasma insulin concentrations in diet-induced obese C57BL/6 J mice. Plasma insulin concentrations at the end of 18 weeks in mice fed diets enriched in lard or saury oil (**a**) and at the end of 8 weeks in mice fed diets enriched in lard, EPA, or LCMUFA (**b**). Values are means ± SEM, *n* = 10. Significantly different mean values (*P* < 0.05) are indicated by different lowercase letters

At 18 weeks, although the plasma glucose levels of mice fed saury oil were not different from those fed the control diet (Control: 18.9 ± 1.6 mmol/L vs. Saury oil group: 16.7 ± 1.1 mmol/L), plasma insulin levels decreased by 38 % (*P* < 0.05) with intake of saury oil (Fig. 6a). At baseline, no differences were found for plasma lipid levels between the control and saury oil groups (Table 4). Although there were no differences in plasma TG levels, mice fed the saury oil diet had significantly lower total cholesterol (TC) (*P* < 0.05), non–HDL cholesterol (non-HDL-C) (*P* < 0.05), and TC:HDL-C ratio (*P* < 0.05) at the 5-, 12-, and 18-week time points compared with control mice.

**Table 4:** Plasma lipid profile at baseline (0 week) and at 5, 12, and 18 weeks of feeding mice diets enriched with (control) or saury oil

	Dietary oils	
Lipid	Lard	Saury oil
0 week	mmol/L	
TG	1.3 ± 0.2	1.1 ± 0.1
TC	2.7 ± 0.2	2.7 ± 0.1
non–HDL-C	1.4 ± 0.1	1.3 ± 0.1
HDL-C	1.3 ± 0.1	1.4 ± 0.1
TC:HDL	2.1 ± 0.1	1.9 ± 0.1
5 weeks		
TG	1.2 ± 0.1	1.0 ± 0.1
TC	4.3 ± 0.3	3.3 ± 0.1**
non–HDL-C	2.2 ± 0.1	1.4 ± 0.1 **
HDL-C	2.1 ± 0.1	1.9 ± 0.1
TC:HDL	2.1 ± 0.1	1.7 ± 0.03*
12 weeks		
TG	0.9 ± 0.2	0.6 ± 0.1
TC	4.5 ± 0.2	3.6 ± 0.1 **
non–HDL-C	2.3 ± 0.2	1.5 ± 0.3 *
HDL-C	2.2 ± 0.1	2.1 ± 0.1
TC:HDL	2.1 ± 0.1	1.7 ± 0.02*
18 weeks		
TG	0.4 ± 0.04	0.4 ± 0.1
TC	4.3 ± 0.3	3.9 ± 0.2
non–HDL-C	2.5 ± 0.2	2.0 ± 0.1*
HDL-C	1.8 ± 0.1	1.9 ± 0.1
TC:HDL	2.4 ± 0.1	2.0 ± 0.1*

Values are means ± SEM, *n* = 10. ^*^ *P* < 0.05, ^**^ *P* < 0.01 compared with control. *TG* triglyceride, *TC* total cholesterol, *HDL-C* HDL cholesterol

#### Liver lipid concentrations

**Fig. 7:**
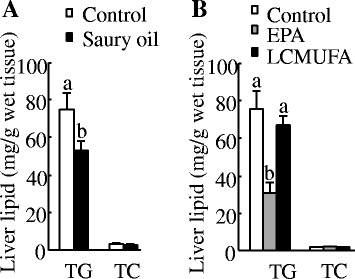
Liver lipid concentrations in diet-induced obese C57BL/6 J mice. Liver lipid concentrations at the end of 18 weeks in mice fed diets enriched in lard or saury oil (**a**) and at the end of 8 weeks in mice fed diets enriched in lard, EPA, or LCMUFA (**b**). Values are means ± SEM, n = 10. Significantly different mean values (*P* < 0.05) are indicated by different lowercase letters

At 18 weeks, although we found no differences in liver weight between the control and saury oil groups (Control: 4.5 ± 0.5 g/100 g liver vs. Saury oil group: 4.5 ± 0.3 g/100 g liver), hepatic TG concentrations were significantly lower by 29 % (*P* < 0.05) in the saury oil group compared with the control (Fig. 7a). This result was further confirmed by Oil Red O staining, which showed a decrease in hepatic lipid deposition in saury oil–fed mice compared with control mice (Additional file 2). Hepatic cholesterol concentrations were not different between the two groups.

#### Adipocyte size

**Fig. 8:**
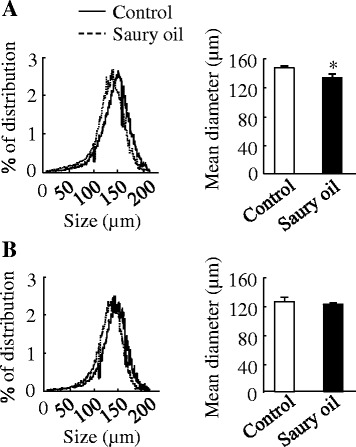
Adipocyte size in diet-induced obese C57BL/6 J mice fed the control or saury oil diet for 18 weeks. Adipocyte size in epididymal adipose (**a**) and subcutaneous adipose (**b**) tissue at the end of 18 weeks. The left panels indicate the frequency distribution of adipocyte size, and the right panels indicate the mean size of adipocytes. Values are means ± SEM, *n* = 4. *P < 0.05 vs. control group

At 18 weeks, the mean adipocyte size in epididymal adipose tissue was about 10 % smaller (*P* < 0.05) in the saury oil group compared with the control (Fig. 8a). We found a non-significant decrease (4 %) in the mean adipocyte size in subcutaneous adipose tissue (Fig. 8b).

#### mRNAs transcribed from genes related to insulin signaling and inflammation in WAT

**Fig. 9:**
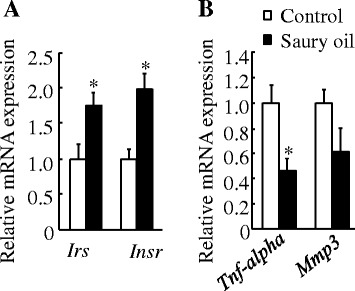
Gene expressions in adipose tissue of diet-induced obese C57BL/6 J mice fed the control or saury oil diet for 18 weeks. mRNAs transcribed from insulin signaling–related genes (**a**) and pro-inflammatory genes (**b**) in epididymal adipose tissue at the end of 18 weeks. Values are means ± SEM, *n* = 10. *P < 0.05 vs. control group

Levels of mRNAs transcribed from *Irs* and *Insr* (Fig. 9a), which are involved in insulin signaling, were significantly increased (*P* < 0.05) by the saury oil diet. mRNA levels of *Tnf-alpha* (Fig. 9b), which are mediators of inflammation, was significantly decreased (*P* < 0.05) by the saury oil diet. We also measured *Mmp3* expression, but there was no significant difference (*P* = 0.15) between the control and saury oil group (Fig. 9b).

### Experiment 2

#### Food intake and body weight

Food intake (control: 2.6 ± 0.4 g/mouse/day vs. EPA group: 2.6 ± 0.2 g/mouse/day vs. LCMUFA group: 2.6 ± 0.3 g/mouse/day) and final body weight (control: 35.1 ± 0.6 g vs. EPA group: 35.2 ± 0.5 g vs. LCMUFA group: 34.9 ± 0.4 g) did not differ among mice fed the control, EPA, or LCMUFA diets. In addition, there were also no significant differences in body weight between the three groups during the 8-week feeding period (Additional file 1).

#### Plasma and organ fatty acid compositions

**Fig. 10:**
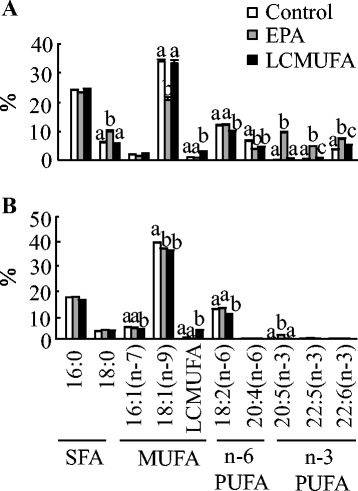
Organ fatty acid compositions in diet-induced obese C57BL/6 J mice fed the control, EPA or LCMUFA diet for 8 weeks. Fatty acid composition in liver (**a**) and epididymal adipose tissue (**b**) at the end of 8 weeks. Values are means ± SEM, *n* = 10. Significantly different mean values (*P* < 0.05) are indicated by different lowercase letters

**Fig. 11:**
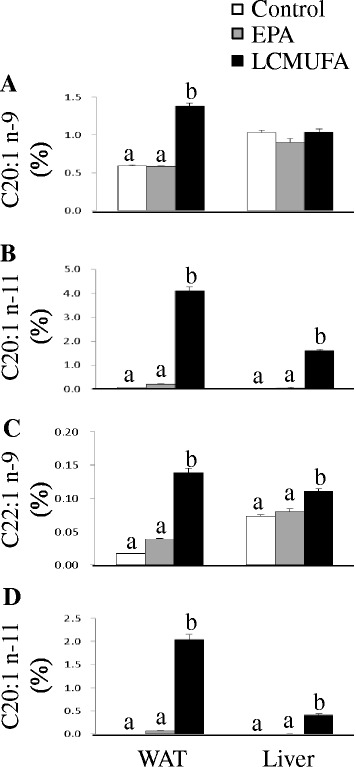
Organ LCMUFA compositions in diet-induced obese C57BL/6 J mice fed the control, EPA or LCMUFA diet for 8 weeks. Percentages of C20:1 n-9 (**a**), C20:1 n-11 (**b**), C22:1 n-9 (**c**) and C22:1 n-11 (**d**) in total lipids of WAT and liver at the end of 18 weeks. Values are means ± SEM, *n* = 10. Significantly different mean values (P < 0.05) are indicated by different lowercase letters. WAT, white adipose tissue

As expected, the fatty acid composition in liver and WAT also reflected the dietary perturbations (Fig. 10). In general, mice fed the EPA diet had proportionally more EPA (~14- to 49-fold) than the control and LCMUFA-fed groups, and proportionally more LCMUFAs (~2- to 9-fold) were found in the LCMUFA group compared with control or EPA groups. For individual types of LCMUFA, there were no significant differences between the control and EPA group. In general, compared with the control or EPA group, each type of LCMUFA had a greater fold change (~1.3 to 205-fold increase) in WAT than in liver (~0.05 to 161-fold increase). Also, as compared with the control or EPA group, the changes in n-11 LCMUFA (C20:1 n-11, ~18 to 161-fold; C22:1 n-11, ~41 to 205-fold increase) were greater than for n-9 LCMUFA (C20:1 n-9, ~0.05 to 1.5-fold; C22:1 n-9, ~0.4 to 12-fold increase) in WAT and liver tissues (Fig. 11). Furthermore, both the EPA and LCMUFA diets decreased the ratios of C16:1/C16:0 (~7–15 %) (*P* < 0.05) and C18:1/C18:0 (~11–33 %) (*P* < 0.05) in WAT as compared with the control group, and the EPA diet decreased the ratio of C16:1/C16:0 by 28 % (*P* < 0.05) and C18:1/C18:0 by 62 % (*P* < 0.05) in the liver (Fig. 5b).

#### Plasma glucose and lipid metabolism

At 8 weeks, plasma insulin levels were significantly (57 %) (*P* < 0.05) lower in the LCMUFA diet group than in the control group, although no statistically significant difference was found between the control and EPA diet groups (Fig. 6b). We found no differences in plasma glucose concentrations among the dietary groups (data not shown). Concerning the plasma lipid profile, the EPA diet decreased plasma TG levels by 49 % (*P* < 0.05) by the end of 4 weeks, and no differences in plasma TG concentrations were found between the control and LCMUFA diet groups (Table 5). Plasma levels of TC, non–HDL-C, and the TC:HDL-C ratio in mice fed the EPA or LCMUFA diets were lower (*P* < 0.05) than in mice fed the control diet at the 4- and 8-week time points. In addition, although EPA consumption significantly decreased (*P* < 0.05) the plasma HDL-C concentration at the 4- and 8-wk time points, we found no difference in plasma HDL-C levels between the control and LCMUFA diet groups.

**Table 5:** Plasma lipid profile at 4 and 8 weeks of feeding mice diets enriched with lard (control), EPA, or LCMUFA

	Dietary oils		
Lipid	Lard	EPA	LCMUFA
4 wk	mmol/L		
TG	0.8 ± 0.1^a^	0.4 ± 0.1^b^	0.9 ± 0.1^a^
TC	4.5 ± 0.1^a^	2.9 ± 0.1^b^	3.6 ± 0.1^c^
non–HDL-C	2.5 ± 0.1^a^	1.1 ± 0.04^b^	1.6 ± 0.1^b^
HDL-C	2.1 ± 0.1^a^	1.8 ± 0.1^b^	2.2 ± 0.1^a^
TC:HDL	2.1 ± 0.1^a^	1.6 ± 0.02^b^	1.6 ± 0.03^b^
8 wk			
TG	0.8 ± 0.2	0.5 ± 0.1	0.6 ± 0.1
TC	4.1 ± 0.1^a^	2.7 ± 0.1^b^	3.7 ± 0.1^c^
non–HDL-C	2.4 ± 0.1^a^	1.1 ± 0.1^b^	1.6 ± 0.1^b^
HDL-C	1.9 ± 0.04^a^	1.6 ± 0.04^b^	2.0 ± 0.1^a^
TC:HDL	2.2 ± 0.1^a^	1.7 ± 0.1^b^	1.9 ± 0.03^c^

Values are means ± SEM, *n* = 10. Means in a row with superscripts without a common letter differ, *P* < 0.05. *TG* triglyceride, *TC* total cholesterol, *HDL-C* HDL cholesterol

#### Liver lipid concentrations

Mice fed the EPA diet had significantly lower (*P* < 0.05) hepatic TG compared with the control or LCMUFA groups (Fig. 7b). Hepatic TG concentrations did not differ between the control and LCMUFA groups. Hepatic cholesterol levels did not differ among the three groups.

## Discussion

The lipid content and fatty acid profile of fish vary largely among species, and consumption of certain species provides high levels of LCMUFAs in addition to n-3 PUFAs. The Pacific saury is common in the diets of East Asians, and the adult saury has a high lipid content (~12–22 %) with concomitantly high levels of long-chain n-3 PUFAs and LCMUFAs. Although many beneficial effects of n-3 PUFA–rich fish oils have been demonstrated, the ability of fish oil rich in LCMUFAs in addition to n-3 PUFAs to attenuate MetS risk factors has not been well studied. Although our previous studies show that saury oil-supplemented diet (4 ~ 6 wk) improved MetS risk factors in MetS mice models, the long-term effect of saury oil on MetS risk factors, energy expenditure, and accumulation of LCMUFA in each organ has not been fully studied yet. Furthermore, very few studies in the literature have compared the effects of EPA and LCMUFA that are enriched in saury oil. In the present study, we demonstrated that feeding with saury oil for 18 weeks ameliorated diet-induced hyperinsulinemia, dyslipidemia, fatty liver, and adipocyte hypertrophy in mice, although the saury oil diet did not alter the energy expenditure. The saury oil diet resulted in significant increases in n-3 PUFAs and LCMUFAs, especially n-11 LCMUFAs, in plasma and vital organs, suggesting a possible involvement of these fatty acids in the beneficial effect of saury oil on MetS. In fact, compared with the control diet, EPA ingestion attenuated hyperlipidemia and hepatic fat accumulation, and LCMUFA ingestion improved hyperinsulinemia and hyperlipidemia in the same mouse model of MetS. In addition, the EPA-rich diet resulted in a significant increase in EPA mainly in liver, and the LCMUFA-rich diet resulted in a significant increase in LCMUFA, especially n-11 LCMUFA, mainly in WAT. The accumulation of EPA and LCMUFA in different organs suggested their possible individual biological effects for improving MetS.

Many features of MetS are associated with insulin resistance, which is related to hyperinsulinemia. A previous study in mice infused with insulin suggested that basal hyperinsulinemia also leads to generalized insulin resistance [15]. In the current study, consumption of both saury oil and an LCMUFA concentrate improved hyperinsulinemia in obese mice; these data are consistent with our previous research, which indicated a beneficial effect of LCMUFA-rich oil on insulin sensitivity in the MetS mouse model [7–9]. Dyslipidemia, which is also a consequence of insulin resistance, is characterized by increased blood levels of LDL-C, reduced HDL-C, and increased TGs. High serum cholesterol is considered to be a strong risk factor for atherosclerosis, and cholesterol-lowering treatment is the gold standard preventive strategy for cardiovascular disease [16]. Furthermore, elevated non–HDL-C signifies an increased risk of cardiovascular disease, and the TC:HDL-C ratio is a reliable measure of cardiovascular disease [17, 18]. Although intake of saury oil did not alter plasma HDL-C and TG levels after 18-week feeding, plasma TC, non–HDL-C levels, and the TC:HDL-C ratio tended to decrease compared with the control diet group. Noteworthily, n-3 PUFA-rich oils generally are supposed to reduce elevated plasma TG levels in human and rodents [19], but we did not observe changes in plasma TG concentration between the control and saury oil groups, probably due to the small dose of n-3 PUFA contained in saury oil (equivalent to ~2 % of EPA plus DHA). Similarly, Mori et al. also reported that no plasma TG changes were observed even when used a relatively high dose of n-3 PUFA-rich fish oil (equivalent to ~4 % of EPA plus DHA) in an experiment feeding mice a high-fat diet for 5 months [20]. Similar plasma lipid patterns were observed in mice fed EPA or LCMUFAs for 8 wk. Relative to the SFA-rich lard diet, both dietary EPA and LCMUFA decreased plasma cholesterol, and EPA lowered non–HDL-C more than LCMUFA did. This finding confirms previous observations that consumption of PUFA or MUFA can lower plasma cholesterol concentrations more so than can SFA [21]. Our results are also consistent with studies feeding mice with either a high fat diet or a high fat diet supplemented with n-3 PUFA-rich fish oil, in which fish oil (equivalent to ~4 % of n-3 PUFA) significantly decreased plasma cholesterol levels. The mechanisms underlying the hypocholesterolemic effect of dietary PUFAs or MUFAs may include upregulation of activity or mRNA levels of the LDL receptor, sterol regulatory element–binding protein 2, and microsomal triglyceride transfer protein [22, 23]. In addition, it is noteworthy that in contrast to EPA, which decreased plasma TC with a concomitant decrease in HDL-C, dietary LCMUFAs did not decrease HDL-C, which is consistent with our previous study [7]. le Morvan et al. reported that an n-3 PUFA–enriched diet decreases plasma HDL-C levels in mice, probably owing to an increase in the amount of scavenger receptor class B-1 in liver [24]. A human study also demonstrated different responses of HDL-C to diets of n-3 PUFA–rich tuna and n-3 PUFA– and LCMUFA-rich salmon [25]. Although the lipoprotein response to LCMUFAs has not been well studied, the shorter-chain MUFA 18:1(n-9) decreases plasma LDL-C levels without lowering HDL-C when fed to humans [26, 27].

Studies have demonstrated that a high-fat diet triggers lipid deposits in liver, and the intrahepatic fat content appears to be tightly related to MetS [28]. Consistent with our histologic observations, biochemical analysis showed that the TG level was greatly reduced in the livers of saury oil–fed mice compared with those of mice fed the lard diet. Furthermore, feeding mice with EPA, but not LCMUFA, significantly decreased hepatic TG concentration. Our study is consistent with the study of Sato et al. [29] who reported that dietary supplementation with EPA strongly suppresses obesity-related elevated hepatic TG content and lipogenic enzymes. Reductions in mRNA and the active protein of sterol regulatory element binding protein-1c (SREBP-1c) may be one of the major mechanisms responsible for the effect of EPA on improving fatty liver [30]. Stearoyl-CoA desaturase-1 (SCD-1) catalyzes the synthesis of MUFAs from saturated fatty acids. The MUFA to saturated fatty acid ratio, particularly C16:1/C16:0 and C18:1/C18:0, affects membrane phospholipid composition, and studies have indicated that alteration in this ratio has been involved in multiple MetS-related diseases, such as diabetes and cardiovascular disease [31] Therefore, the activity of SCD-1 is important in MetS states. Decreased SCD-1 activity is actually associated with improved adiposity and insulin sensitivity, and n-3 PUFAs inhibit expression of *Scd-1* in mouse liver [32–34]. In the current study, saury oil and EPA significantly decreased SCD-1 desaturation index (C16:1/C16:0 and C18:1/C18:0) in liver and WAT tissues, and LCMUFA decreased these SCD-1 desaturation index only in WAT but not liver. Therefore, the effect of saury oil on improving hepatic steatosis may possibly be attributed to EPA and may partly be due to inhibition of SCD-1 activity. On the other hand, the absence of a change in the desaturation index in liver by dietary LCMUFAs may at least partly explain the lack of improvement in hepatic lipid deposition. This is probably due to a less accumulation of LCMUFA in liver than WAT.

Adipose tissue is a highly active endocrine organ with the capacity to produce various bioactive mediators that modulate hemostasis, lipid and glucose metabolism, and inflammation [35]. Inflammatory cytokines associated with hypertrophic adipocytes may contribute to the development of many aspects of MetS and insulin resistance. The saury oil diet decreased adipocyte size, with concomitant increases in mRNAs transcribed from insulin signaling genes (*Irs* and *Insr*) and decreases in mRNAs transcribed from pro-inflammatory genes (*Tnf-alpha* and *Mmp3*). Thus, the beneficial effects of saury oil on plasma glucose and lipid homeostasis may be partly linked to morphologic and inflammatory changes in adipose tissue. Previous studies have also shown that a diet rich in EPA or LCMUFA reduces adipocyte size and improves adipose inflammation in mice [7, 8, 35]. Because saury oil, EPA, or LCMUFA consumption lowered the ratios of 16:1/16:0 and 18:1/18:0 in adipose tissue, both EPA and LCMUFAs may contribute to the beneficial effect of saury oil on adipocyte hypertrophy that may be partially due to decreased SCD-1 activity.

Obesity-related metabolic diseases are associated with decreased energy expenditure. In the current study, we also observed a significant time-dependent decrease in respiratory quotient and locomotor activity with high-fat diet. A 15 % (w/w) EPA-supplemented diet increases oxygen consumption and improves the respiratory quotient in high-fat diet–induced obese mice [36]. N-3 PUFA–rich saury oil consumption, however, did not alter parameters of energy expenditure in our study. The smaller dose of EPA [1 % (w/w)] in the saury oil diet and the different animal model used in the present study may be possible explanations for these differences.

Our data showed LCMUFA may improve serum lipid levels and potentially prevent diet-induced dyslipidemia, suggesting the potential application of LCMUFA-rich fish oil as nutraceuticals in overcoming the negative effects of lipid disorders. Statin drugs are widely considered as successful lipid-lowering therapeutic agents that reduce the risks associated with cardiovascular disease. However, due to many adverse side effects, such as muscle problems, diabetes and increased cancer risk, alternative lipid-lowering therapy should be considered [37]. Scicchitano et al. have reviewed the role of nutraceuticals and functional foods in the management of dyslipidemia, and showed the beneficial role of fish oil (alone or in combination with statins) as a nutraceutical in dyslipidaemia treatment in both animals and humans [38]. Noteworthily, most fish oils used in these studies were rich in n-3 PUFA, and our findings from animal studies offer new insight into fish oils that are enriched in LCMUFA. Nevertheless, further studies are needed to elucidate the influence of LCMUFA-rich fish oil as nutraceuticals for lipid lowering in humans.

There are some limitations in our study. First, due to lack of a chow-diet control group, we could not quantitatively determine how much saury oil improved MetS. Additionally, while the significant decreases in plasma insulin concentration on the saury oil diet is likely due to a decrease in obese-related insulin resistance, glucose tolerance tests will be necessary in future studies to verify the effects of EPA- and LCMUFA-rich oils on insulin resistance. In addition, because the LCMUFA-rich oil used in the current study is a mixture of LCMUFA isomers, investigation of the effects of specific isomers will be necessary in future studies.

## Conclusion

In our long-term (18 wk) feeding study, saury oil enriched in n-3 PUFA and LCMUFA significantly improved risk factors for metabolic syndrome in diet-induced obese mice, by possible combined effects of EPA and LCMUFA on altering plasma lipid levels, as well as by other mechanisms, such as favourable changes on hepatic lipid deposition, plasma insulin levels, and the fatty acid desaturation index in adipose tissue. Our results suggest that both n-3 PUFA and LCMUFA may contribute to saury oil-mediated improvement of metabolic syndrome with shared and complementary mechanisms.

## Additional files

Additional file 1:
**Body weight gain in diet-induced obese mice.** (DOCX 50 kb) Additional file 2:
**Oil red O staining of liver sections from diet-induced obese mice fed the control diet or saury oil diet for 18 weeks in Experiment 1.** (DOCX 99 kb)
