# Supplementary material for: Long-term dietary supplementation with saury oil attenuates metabolic abnormalities in mice fed a high-fat diet: combined beneficial effect of omega-3 fatty acids and long-chain monounsaturated fatty acids
Source: Lipids Health Dis. 2015 Dec 1;14:155. doi: 10.1186/s12944-015-0161-8 (PMC4666194; doi:10.1186/s12944-015-0161-8)
Supplement: Additional file 1: — Body weight gain in diet-induced obese mice. (DOCX 50 kb) [file 12944_2015_161_MOESM1_ESM.docx]

**Additional file 1 – Body weight gain in diet-induced obese mice.**


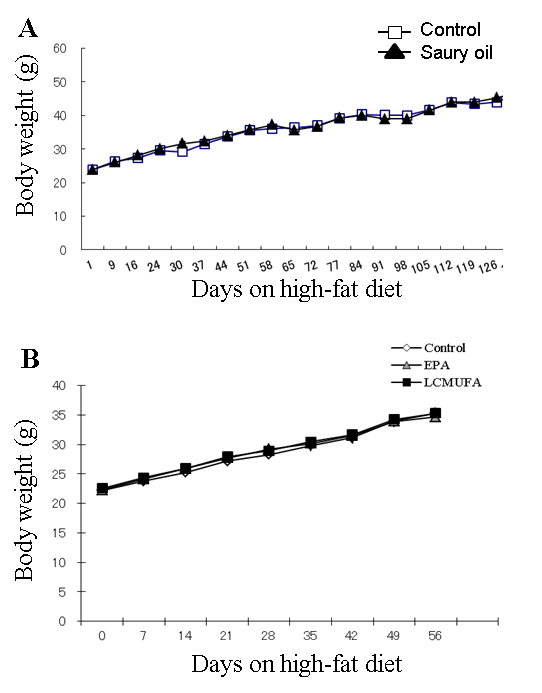


Body weight change in diet-induced obese mice fed the control diet or saury oil diet for 18 weeks in Experiment 1 (A), and in diet-induced obese mice fed the control diet, EPA diet, or LCMUFA diet for 8 weeks in Experiment 2 (B).
