# Supplementary material for: Long-term dietary supplementation with saury oil attenuates metabolic abnormalities in mice fed a high-fat diet: combined beneficial effect of omega-3 fatty acids and long-chain monounsaturated fatty acids
Source: Lipids Health Dis. 2015 Dec 1;14:155. doi: 10.1186/s12944-015-0161-8 (PMC4666194; doi:10.1186/s12944-015-0161-8)
Supplement: Additional file 2: — Oil red O staining of liver sections from diet-induced obese mice fed the control diet or saury oil diet for 18 weeks in Experiment 1. (DOCX 99 kb) [file 12944_2015_161_MOESM2_ESM.docx]

**Additional file 2 – Oil red O staining of liver sections from diet-induced obese mice fed the control diet or saury oil diet for 18 weeks in Experiment 1.**


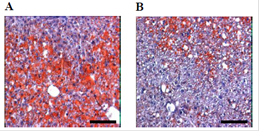


Representative photomicrographs of liver sections stained with Oil red O in control (A) or saury oil group (B). 1. Scale bars: 100 µm
